# Preparation of Flexible Calcium Carbonate by In Situ Carbonation of the Chitin Fibrils and Its Use for Producing High Loaded Paper

**DOI:** 10.3390/ma16082978

**Published:** 2023-04-09

**Authors:** Sang Yun Kim, Sun Young Jung, Yung Bum Seo, Jung Soo Han

**Affiliations:** 1 Department of Bio-Based Materials, Chungnam National University, Yousung-Gu, Daejeon 34134, Republic of Korea; 2 Institute of Agricultural Science, Chungnam National University, Yousung-Gu, Daejeon 34134, Republic of Korea

**Keywords:** chitin microfibril, cellulose nanofibril, flexible calcium carbonate (FCC), highly loaded paper, paper bulk

## Abstract

Flexible calcium carbonate (FCC) was developed as a functional papermaking filler for high loaded paper, which was a fiber-like shaped calcium carbonate produced from the in situ carbonation process on the cellulose micro-or nanofibril surface. Chitin is the second most abundant renewable material after cellulose. In this study, a chitin microfibril was utilized as the fibril core for making the FCC. Cellulose fibrils for the preparation of FCC were obtained by fibrillation of the TEMPO (2,2,6,6-tetramethylpiperidine-1-oxyl radical) treated wood fibers. The chitin fibril was obtained from the β-chitin from the born of squid fibrillated in water by grinding. Both fibrils were mixed with calcium oxide and underwent a carbonation process by the addition of carbon dioxide, thus the calcium carbonate attached on the fibrils to make FCC. When used in papermaking, both the FCC from chitin and cellulose gave a much higher bulk and tensile strength simultaneously than the conventional papermaking filler of ground calcium carbonate, while maintaining the other essential properties of paper. The FCC from chitin caused an even higher bulk and higher tensile strength than those of the FCC from cellulose in paper materials. Furthermore, the simple preparation method of the chitin FCC in comparison with the cellulose FCC may enable a reduction in the use of wood fibers, process energy, and the production cost of paper materials.

## 1. Introduction

Inorganic fillers such as calcium carbonate (CaCO_3_) are used as alternatives to the much more expensive wood fibers in paper and may reduce the material cost as well as the process cost such as the drying energy. Increasing the filler content in paper is also very important for a reduction in the production cost, but increases of only 1–2% of filler content in the paper have been limited due to the loss of essential properties such as strength, bulk, and stiffness [1,2]. Up until now, papermakers worldwide have tried to increase the filler content in paper. The negative effect of the filler in the paper strength property is mainly caused by the fact that the filler interrupts the fiber–fiber bonding between wood fibers, so many high loading technologies have been developed by focusing on reducing the surface area of the filler to allow for more fiber–fiber bonding in the paper.

The pre-flocculation of fillers by making flocs of precipitated calcium carbonate (PCC) or ground calcium carbonate (GCC) by using electrolyte polymers is a well-known high-loading technique [3,4,5,6,7]. However, pre-flocculation is limited to improving the bulk (cm^3^/g) of paper, which is one of the critical properties.

We have developed various types of functional fillers for high loading by utilizing an in situ CaCO_3_ formation process. Hybrid calcium carbonate (HCC) and post hybrid calcium carbonate (pHCC) were developed by combining the pre-flocculation and in situ carbonation process to produce the semi-rigid CaCO_3_ flocs [2,8,9,10,11,12,13]. It was demonstrated that the paper containing 40% HCC had equivalent physical properties to the paper containing 30% GCC [8].

The other type of filler is flexible calcium carbonate (FCC) [14,15]. FCC is made by forming CaCO_3_ on the surface of cellulose nano- or microfibrils (CNF or CMF) through an in situ CaCO_3_ formation method where the weight ratios of the newly formed CaCO_3_ to CNFs varied from 40:1 to 80:1. It has a large size and is flexible and deformable due to the CNF core, which could simultaneously provide the extreme bulk and strength of paper, and developed high smoothness after calendaring.

Many studies have discussed using CNF or CMF as the filler-modifying material [16,17,18]. Like FCC, they could improve the strength properties and smoothness of the paper, but most of them failed to improve the paper bulk, which is one of the critical properties of paper.

FCC has been made from cellulose micro- or nanofibrils, which were prepared from the bleached kraft pulp using only mechanical fibrillation [14,15]. Investigation of other sources for the fibril core of the FCC is important for developing a more cost-effective FCC process. For example, recycled fibers could be a candidate such as post-consumer old corrugated container (OCC) [19].

Chitin is the second most abundant biopolymer, occurring mainly in the exoskeletons of shellfish, insects, and mushrooms [20]. Like cellulose, chitin exists as a hierarchical structure consisting of a bundle of nanofibrils in the exoskeleton of arthropods. Therefore, chitin micro-or nanofibrils can be extracted through mechanical treatment [21].

In this study, we tried to utilize the chitin microfibril (ChMF) as the source of the fibril core to prepare the FCC. Chitin was fibrillated to chitin microfibrils using an ultra-fine grinding machine and treated with an in situ CaCO_3_ formation process to make the chitin-based FCC (chitin-FCC). The properties of the chitin-FCC-containing handsheet paper were compared with the GCC and cellulose-based FCC. Especially for the cellulose-based FCC (TEMPO-FCC), CNF made by 2,2,6,6-tetramethylpiperidine-1-oxyl radical (TEMPO) mediated oxidation (TEMPO-CNF) was used [22].

This study examined the change in the characteristics of the FCC prepared from the different types of fibrils and the physical properties of its-filled paper as well as the potential of the chitin for making a high-loading papermaking filler for savings in production and energy costs and wood resources in the paper industry.

## 2. Materials and Methods

### 2.1. Materials and Handsheet Preparation

The ground calcium carbonate (GCC) was kindly supported by Omya Korea Inc., located in Jaecheon, South Korea, of which the mean size was reported to be 2.0 μm. Calcium oxide was purchased from Korea Showa Chemicals Co. (Seoul, South Korea). As a retention aid for papermaking, cationic polyacrylamide (C-PAM, molecular weight 5–7 million g/mol, +5 meq/g) from CIBA Specialty Chemical Korea (Basel, Switzerland) was used at 0.1% based on the dry weight of the papermaking furnish. Handsheet preparation was carried out according to the standard method (TAPPI T205 sp-95). To make the handsheets containing fillers, we used a mixture (20:80) of commercial softwood bleached Kraft pulp (SwBKP; a mixture of hemlock, Douglas fir, and cedar) and hardwood bleached Kraft pulp (HwBKP; a mixture of aspen and poplar), respectively, as the wood fiber furnish, both of which came from Canada. These wood pulps were refined together in a valley beater until their freeness reached 500 mL CSF (TAPPI T227 om-99). Then, after mixing the fibers and fillers to make handsheets with ash contents of 30 and 40 wt.%, we prepared handsheets of 60 g/m^2^ basis weight (TAPPI T205 sp-95). It was difficult to meet the exact filler content of 30 and 40 wt.%; however, once the filler content was closely fixed to the target content, there was almost no variation in the filler content per each type of filler by making handsheets with the same amount of furnish. In the case of chitin-based flexible calcium carbonates (chitin-FCCs), we made another type of handsheet by adding 3% cationic starch based on the dry weight of the furnish for strength supplementation (degree of substitution (DS) 0.037, Suncasta 6020, Samyang, Seoul, Korea). The ash content (TAPPI 413 om-93), bulk (TAPPI T411 om-97), tensile strength (ISO 1924), Bekk smoothness (TAPPI T479 cm-99), ISO brightness (ISO 2470), opacity (ISO 2471), and Gurley stiffness (TAPPI T543 om-00) of the handsheets were measured according to the standard methods.

### 2.2. Preparation of Chitin Microfibril (ChMF)

The chitin type for producing chitin microfibril (ChMF) was β-chitin. β-Chitin, purified from the Loligo Vulgaris squid (i.e., squid pen), was purchased from Marin Bo Resources Co., Ltd., Samutsakhon, Thailand. The degree of substitution (DS) value of the acetyl groups of the chitin was calculated from the C and N content in the elemental analysis data obtained using an elemental analyzer (FLASH 2000, Thermo Fisher Scientific, Waltham, MA, USA) according to the method in [23]. The calculated DS value of chitin was 0.98.

Chitin was pulverized by a kitchen blender and fibrillated to make ChMF by using Super Masscolloider (Masuko Sangyo Co., Ltd., Kawaguchi, Japan), an ultra-fine grinding machine. The pulverized chitin suspension of 1 wt.% concentration in water was repeatedly passed through the grinding machine with up to 10 passes at 1500 rpm. The gap between the two grinding stones was kept at −100 μm from the zero position by controlling the bottom grinding stone after the sample was loaded.

The cellulose nanofibrils (CNF, TEMPO-CNF) used in this study were kindly donated by a paper mill located in Daejun, Republic of Korea. They were prepared by 2,2,6,6-tetramethylpiperidine-1-oxyl radical (TEMPO) mediated oxidation and subsequent disintegration by a high-pressure homogenizer according to the method published [22]. We measured the dimensions of the prepared ChMF and TEMPO-CNF using a scanning electron microscope (SEM, S-4800, Hitach, Tokyo, Japan) and transmission electron microscope (TEM, JEM-2100, JEOL, Tokyo, Japan), respectively.

### 2.3. Preparation of Flexible Calcium Carbonate (FCC)

Flexible calcium carbonates (FCCs) from ChMF and TEMPO-CNF were prepared by an in situ carbonation process according to [14,15]. To prepare the chitin-FCCs, the consistency of the ChMF suspension was controlled to 0.2% at 30 °C. The weight ratios of CaCO_3_ to ChMF were 40.0 and 80.0, and they were named as chitin-FCC 40 and chitin-FCC 80, respectively. In more detail, one liter of ChMF suspension in a 2 L beaker had 2 g ChMF (dry weight), and the desired amount of calcium oxide (44.9 g for chitin-FCC 40, 89.9 g for chitin-FCC 80) were added to produce CaCO_3_ on the surface of ChMF (80 g for chitin-FCC 40, 160 g for chitin-FCC 80) by injecting carbon dioxide (flow rate 3 L/min) to the suspension. Chitin-FCC 80 only needed 50% of ChMF when compared to chitin-FCC 40. If chitin-FCC 80 has equivalent properties in paper to chitin-FCC 40, the production cost of chitin-FCC should be lowered. The temperature was held at 30 °C initially and raised by the exothermic reaction to 33–34 °C at the termination of reaction while stirring. When the pH reached 7.0, we waited two more minutes to confirm the stable pH and terminated the reaction.

The CNF-based FCC (TEMPO-FCC) was prepared by the same method as chitin-FCC. For the TEMPO-FCC from TEMPO-CNF, the weight ratio of CaCO_3_ to CNF was fixed to 40.0. We named this TEMPO-FCC as TEMPO-FCC 40. The morphologies of the prepared FCCs were analyzed using SEM. Attenuated total reflectance-Fourier transform infrared spectroscopy (FTIR, Nicolet Nexus 670, Thermo Fischer, USA) was performed to characterize the chemical structure of CaCO_3_ in the FCCs.

## 3. Results

### 3.1. Chitin Fibrils as an Alternative for Cellulose Fibrils

Han et al. (2020) [14] and Kim et al. (2022) [15] demonstrated that FCCs from highly fibrillated cellulose (CMF and CNF) could provide a remarkable improvement in both the bulk and strength properties of paper. However, the high production cost of the fibrillated cellulose may be a challenge in producing FCC. The energy-intensive fibrillation process is the main reason for its high production cost [24]. The use of expensive wood fibers as the raw material also makes up a large portion of the production cost [25]. Furthermore, wood cutting for the procurement of wood fibers has been recognized to have a negative impact on the environment, although modern paper mills use FSC (Forest Stewardship Council)-certified pulp, which means that the trees for pulp production are harvested responsibly so that there is no net loss of forest over time [26].

Alternatives for the raw materials used to produce fibril substrates are desirable. Chitin, one of the most abundant biomasses on Earth, was used in this study. Squid pens, which consist of chitin, are considered as waste by the fishery industry [27]. Squid pens are an important source of β-chitin, one of the chitin crystal types. Unlike α-chitin, another type of chitin produced from crab and prawn shells, β-chitin does not demand a hydrochloric acid treatment for demineralization because squid pens contain few calcium salts and pigments, which is an advantage from the environment perspective as well as in terms of the process cost for chitin fibril production [28]. Additionally, it turned out that the chitin could be easily fibrillated to the micro- and nano-fibrils with less energy consumption than wood fibers.

### 3.2. Morphology of Chitin Microfibril and Flexible Calcium Carbonate

**Figure 1:**
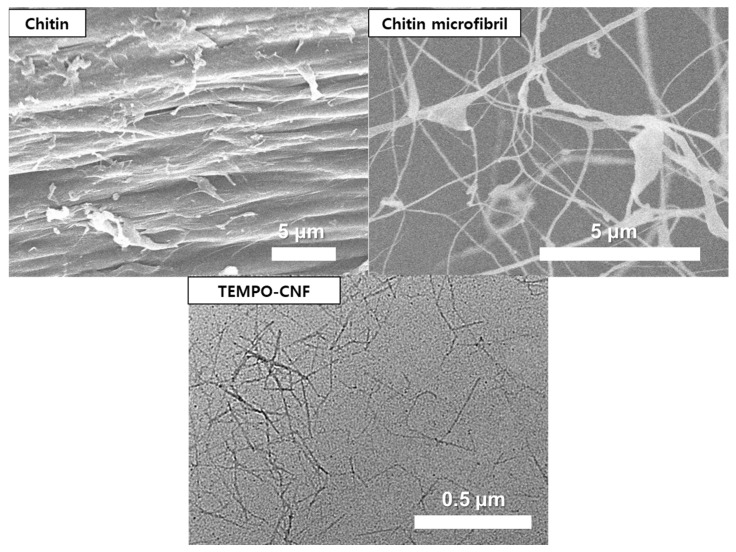
The SEM images of the chitin, chitin microfibril produced from it, and the TEM image of TEMPO-CNF.

The micrographs of chitin, prepared ChMF, and TEMPO-CNF are shown in Figure 1. The average dimension of the ChMF used in this study was 50~100 nm in width and several microns in length. We found that the ChMF in this study had a similar morphology to the CMF used in the previous study conducted by our group [14]. By using the same grinder, the passes for wood fibers were 30 times, and for the chitin used in this study, 10 times. This means that the chitin fibrillation needed less energy than the wood fiber fibrillation.

We used TEMPO-CNF in this study as the cellulose fibril core to prepare CNF-based FCC (TEMPO-FCC) for a comparison with the chitin-based FCC (chitin-FCC). TEMPO-CNF had a very small width under 5 nm and a length of about 1 μm. To prepare the FCCs, a high amount of CaCO_3_ (40 times by CNF weight) was attached on the surface of fibrils by an in situ carbonation process. We believe that the specific surface area of the fibril is highly related to the amount of CaCO_3_ attached.

**Figure 2:**
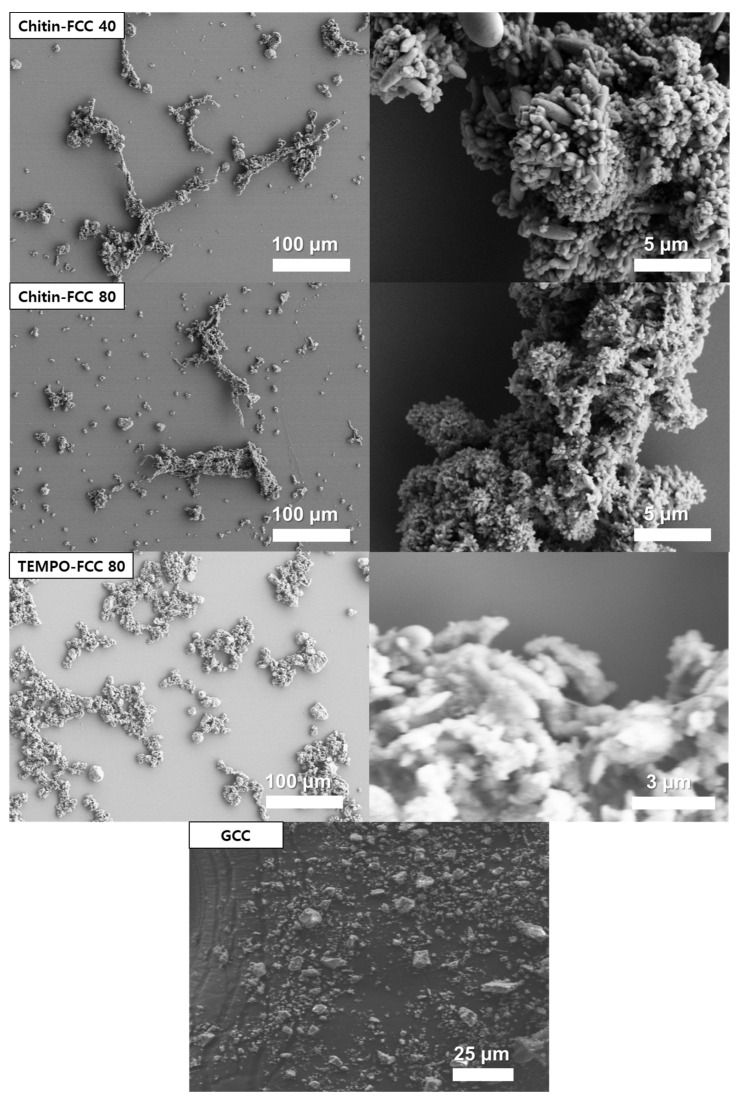
Micrographs of the FCCs from the chitin microfibril (chitin-FCC 40 and 80), and TEMPO-CNF (TEMPO-FCC 40) at low magnification (**left**) and high magnification (**right**). The GCC (2 μm) micrograph is also shown as a reference.

The morphology of chitin-FCC, TEMPO-FCC, and GCC is presented in the micrograph in Figure 2. As seen in Figure 2, very small-sized CaCO_3_ particles were distributed all over the micrographs in the GCC case. FCCs from ChMF (chitin-FCC 40 and 80) exhibited a similar morphology to the FCC from TEMPO-CNF in that they had fiber-like shapes

**Figure 3:**
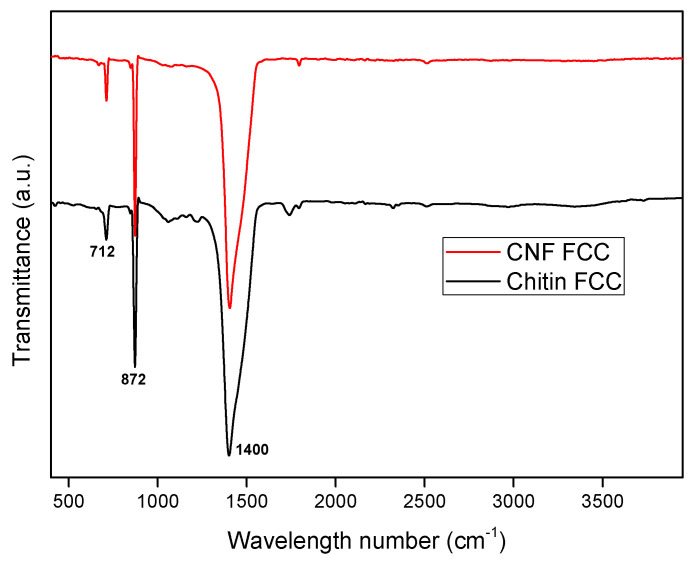
FTIR spectra of the prepared FCCs from ChMF and CNF.

High magnification images indicated that both FCCs from ChMF and TEMPO-CNF consisted of the scalenohedral type of CaCO_3_. This scalenohedral type CaCO_3_ was calcite because it showed characteristics peaks indicating the calcite CaCO_3_ in the ATR-IR results in Figure 3 [29].

Chitin-FCC 40 did not have very small-sized particles. We believe that most of the CaCO_3_ formed by the in situ carbonation process was attached to the surface of ChMF and TEMPO-CNF. However, for chitin-FCC 80, with the weight ratio of CaCO_3_:ChMF of 80:1, small-sized CaCO_3_ particles began to be observed. This means that some of the CaCO_3_ could not be formed on the surface of ChMF in the chitin-FCC 80 case. As above-mentioned, ChMF had a similar dimension with CNF in our previous study, where FCC with the weight ratio of CaCO_3_:CNF of 80:1 could successfully be made without any detached small CaCO_3_ particles [14]. Therefore, this occurrence of small CaCO_3_ particles in chitin-FCC 80 might not be from the surface area effect, but from the surface chemical difference between cellulose and chitin.

Chitin is the cellulose analog, but it consists of N-acetyl glucosamine as the repeating sugar. The degree of substitution (DS) values of the acetyl group of chitins used in this study was calculated to be 0.90, indicating that chitin had less hydroxyl groups than cellulose. The hydroxyl group of the polymer like cellulose can act as the nucleating site for growing the CaCO_3_ [30]. Therefore, in chitin-FCC 80, the chitin could not attach all the formed CaCO_3_ on its surface due to there being less hydroxyl groups to attach the CaCO_3_ than that of cellulose, but it should be studied in detail in the future.

The FCC from TEMPO-CNF and TEMPO-FCC 80 also had long CaCO_3_ agglomerates larger than the GCC, but smaller than those of chitin-FCC 40 and 80. This could be attributed to the dimensions of TEMPO-CNF being lower than those of ChMF. Han et al. [14] reported that the prepared FCC size was dependent on the size (degree of the fibrillation) of the fibrils. A few small sized-CaCO_3_ particles were observed, which indicates that the high surface area of TEMPO-CNF was enough to attach a large amount of CaCO_3_ to the surface of the fibril.

### 3.3. Physical Properties of Paper

**Figure 4:**
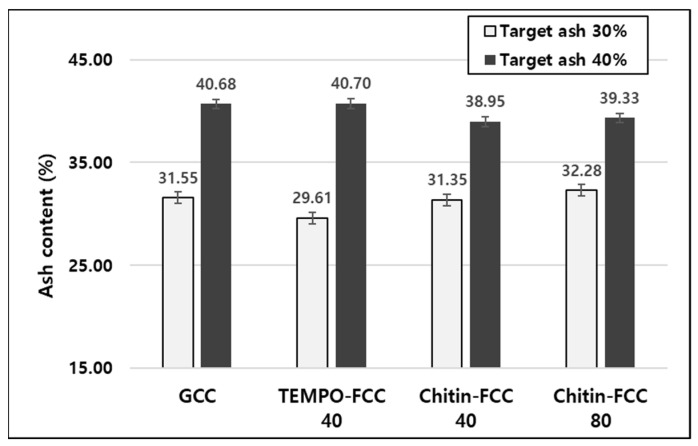
Ash content of the papers containing three different fillers.

The FCCs and GCC were added to the papermaking furnish in two different target ash contents. The ash contents of the papers are presented in Figure 4. The ash contents of four different filler-containing papers were very close to the target filler contents. Therefore, we decided to compare the physical properties of the papers containing different fillers at each target ash content.

**Figure 5:**
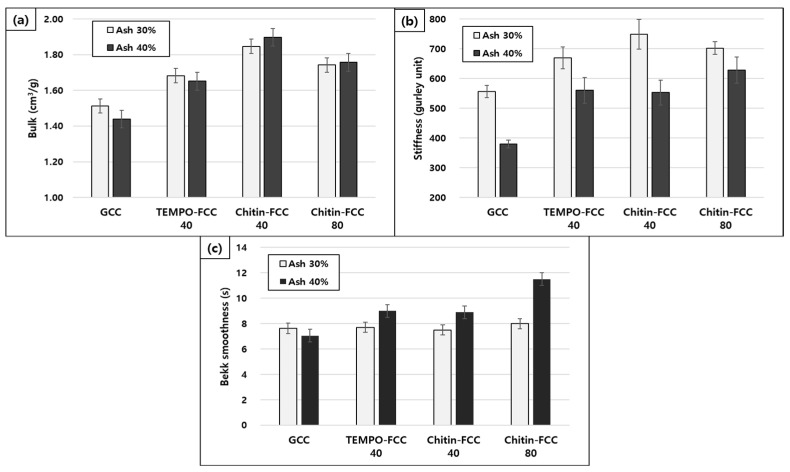
Bulk (**a**), Gurley stiffness (**b**), and Bekk smoothness (**c**) of the papers containing three different fillers.

The bulk, stiffness, and Bekk smoothness of papers containing four different fillers are presented in Figure 5. All FCCs improved the bulk of their filled paper remarkably more than the GCC (Figure 5a). The high bulk was due to the larger size of the FCC than that of GCC, as shown in the micrographs in Figure 2.

TEMPO-FCC 40 showed slightly less improvement in the bulk than chitin-FCCs. In the chitin-FCCs, paper containing chitin-FCC 80 had a lower bulk than one containing chitin-FCC 40. Chitin-FCC 80 had small-sized CaCO_3_ particles as well as large-sized FCCs. The portion of the small-sized CaCO_3_ particles in chitin-FCC 80 would not contribute to the improvement in the bulk. The stiffness of paper is strongly dependent on the paper thickness (or bulk). Indeed, as presented in Figure 5b, the trend of the stiffness results of the papers was the same as that of the bulk results.

The Bekk smoothness of papers is presented in Figure 5c. In this study, with the 30% ash content, the smoothness values of the paper containing FCCs were similar to the GCC-containing paper, and higher with the 40% ash content. The deformability of FCC inside the paper in the wet pressing process could explain this result [14,15]. The highest Bekk smoothness value of chitin-FCC 80 with a 40% ash content could be attributed to the portion of the small-sized CaCO_3_.

**Figure 6:**
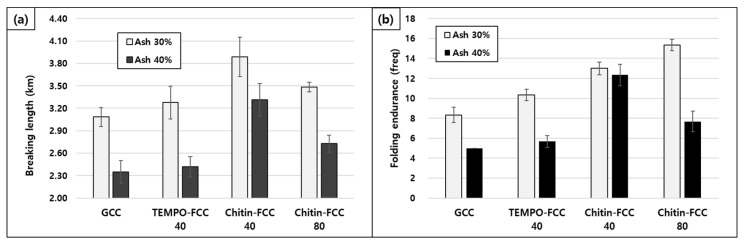
Breaking length (**a**) and folding property (**b**) of the papers containing different fillers.

The tensile strength and folding properties of papers containing FCCs and GCC are presented in Figure 6. In Figure 6a, the tensile strength of paper is exhibited as the breaking length, which is a measure of tensile strength after compensating basis weight differences. The breaking lengths of the chitin-FCC40 papers showed the highest values among the papers with the same ash contents. In particular, the breaking length difference increased with the 40% ash content. The higher strength of FCC than that of GCC was attributed to the lower specific surface area of FCC due to its large size and the presence of a lower amount of small particles in FCC [14]. Therefore, the lower strength of chitin-FCC 80 than that of chitin-FCC 40 could be due to the presence of small-sized CaCO_3_ particles. In the case of TEMPO-FCC 40, the breaking length of its containing paper was lower than that of chitin-FCC 40. We do not know the exact reason as to why the TEMPO-FCC had a lower breaking length than that of chitin-FCC yet. The effect of the fibril core material properties on developing the bonding properties of FCC in paper should be investigated in the follow-up study.

The folding endurance is related to the paper cracking during folding. The paper containing FCCs exhibited higher folding endurance values than that of GCC. With a 40% ash content, chitin-FCC 40 showed the highest value. This behavior was related to the breaking length, but it should be studied in detail in the future.

### 3.4. Trials to Save Wood Fiber

**Figure 7:**
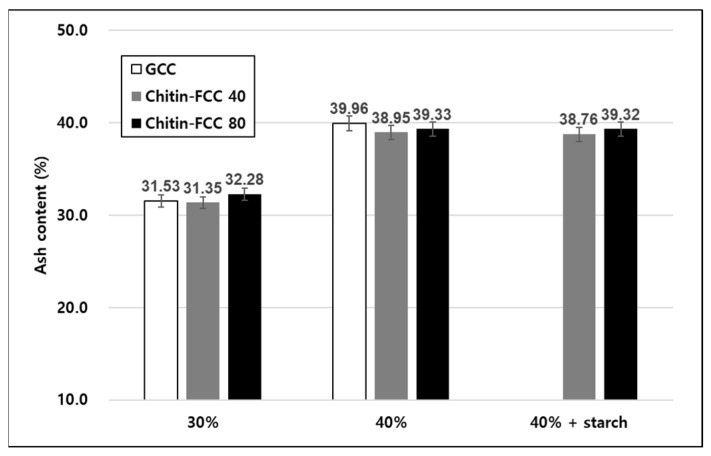
Experimental set of another type of handsheet with the ash content of the papers containing GCC, chitin-FCC, and chitin-FCC with starch.

**Figure 8:**
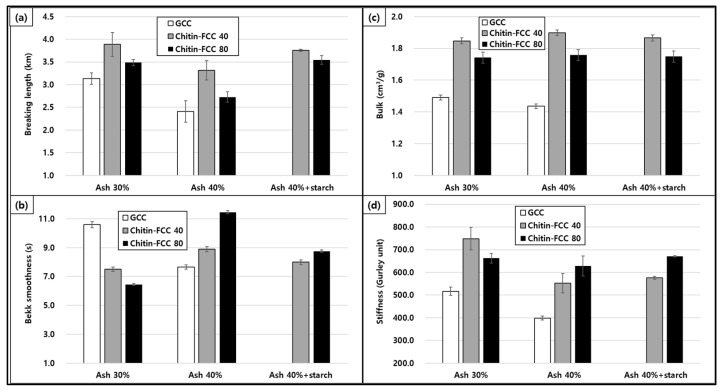
Paper properties including breaking length (**a**), smoothness (**b**), bulk (**c**), and stiffness (**d**) of the papers containing GCC, chitin-FCC, and chitin-FCC with starch.

To utilize the advantage of chitin-FCCs for improving paper properties, starch was added by 3 wt.% to the paper furnish for strength supplementation. The ash contents and physical property results are presented in Figure 7 and Figure 8. As presented in Figure 7, the ash contents of papers containing different fillers were close to the target filler contents regardless of the starch addition. Therefore, we could compare the paper properties at each target ash content. As presented in Figure 8a, through the addition of starch, the paper with chitin-FCC 40 and chitin-FCC 80 could have a better breaking length than the paper with GCC 30% while chitin-FCC 40 without starch still had an equivalent breaking length to GCC 30%. This indicates that chitin-FCCs showed the potential to save wood fibers by about 10% while maintaining competent properties. Less use of wood fibers would reduce the papermaking costs in terms of the wood fiber cost and drying energy expense.

## 4. Discussion

Chitin, the second most abundant biopolymer after cellulose was used to make a chitin-based flexible calcium carbonate (chitin-FCC). Chitin was mechanically fibrillated to produce chitin microfibrils and used as the fibril core of the chitin-FCC by attaching CaCO_3_ on the surface of the chitin microfibril by the in situ carbonation process (weight ratio of CaCO_3_:chitin microfibril of 40:1 for chitin-FCC 40 and 80:1 for chitin-FCC 80, respectively). It was found that the mechanical fibrillation energy for the preparation of chitin microfibrils was much less than that of the cellulose nanofibril from wood fibers. Chitin-FCC 40 from chitin had a similar morphology to TEMPO-FCC 40 from cellulose where the cellulose fibrils generated by the application of TEMPO were used to produce FCC. Both FCCs showed remarkably higher physical properties than GCC when used in papermaking, and chitin-FCC 40 provided higher bulk and strength properties than TEMPO-FCC40. We believe that the material property difference between chitin and cellulose caused these differences. Even in the case of chitin-FCC 80, which contained half the amount of fibrils than chitin-FCC 40, its paper properties were equivalent to those of TEMPO-FCC 40. It was demonstrated that 10% more fillers could replace the wood fibers when GCC was replaced by either chitin-FCC 40 or chitin-FCC 80. However, in the chitin-FCC 80 case, the addition of 3% starch was necessary to supplement the low tensile strength.

It was found that the chitin from squid pens could replace the cellulose from wood fibers when producing FCC, and all the benefits from FCC from cellulose could be realized without the negative effects.

## Data Availability

Not applicable.
